# Apigenin, a Single Active Component of Herbal Extract, Alleviates Xerostomia *via* ERα-Mediated Upregulation of AQP5 Activation

**DOI:** 10.3389/fphar.2022.818116

**Published:** 2022-02-21

**Authors:** Wei Wei, Tingting Cao, Janak L. Pathak, Xintong Liu, Tianjiao Mao, Nobumoto Watanabe, Xiaomeng Li, Manli Zhang, Jiang Li

**Affiliations:** ^1^ The Key Laboratory of Molecular Epigenetic, Institute of Genetics and Cytology, Northeast Normal University, Changchun, China; ^2^ Guangdong Engineering Research Center of Oral Restoration and Reconstruction, Affiliated Stomatology Hospital of Guangzhou Medical University, Guangzhou, China; ^3^ Chemical Biology Research Group, RIKEN Center for Sustainable Resource Science, Saitama, Japan; ^4^ Bio-Active Compounds Discovery Unit, RIKEN Center for Sustainable Resource Science, Saitama, Japan; ^5^ Hospital of Stomatology, Jilin University, Changchun, China

**Keywords:** apigenin, xerostomia, aquaporin5 (AQP5), estrogen receptor *α* (ERα), ovariectomized (OVX) mice

## Abstract

Xerostomia is a common symptom in menopausal women, suggesting the role of sex steroids in disease development. Shreds of literature had reported the potential use of herbal extracts to relieve xerostomia. However, a cocktail of multiple components in herbal extract makes it difficult to understand the exact mechanism of action. Aquaporin5 (AQP5), the specific aquaporin expressed in salivary glands, plays an important role in salivary secretion as a downstream of estrogen signaling. In this study, we aimed to unravel a single active herbal component as a therapeutic for xerostomia and investigate its mechanism of action. The effects of apigenin (flavonoid), dauricine (alkaloids), protopine (alkaloids), and lentinan (polysaccharides) on AQP5 transcription were screened *in vitro*. Only apigenin robustly induced AQP5 transcription and expression, and this effect was even robust compared to the effect of estradiol (E2, a positive control). Overexpression of estrogen receptor *α* (ERα) in the human salivary gland cell line (HSG) upregulated the AQP5 transcription and expression and the knockdown ERα reversed this effect, suggesting the role of ERα signaling on AQP5 activation in HSG cells. Docking results showed apigenin-specific binding sites in ERα. We further analyzed the therapeutic effect of apigenin on ovariectomized mice as a xerostomia model. The saliva secretion in the xerostomia group was reduced to one-third of the sham group, whereas the apigenin or E2 treatment for 12 weeks reversed this effect. Meanwhile, the water consumption in the xerostomia group was augmented obviously compared to the sham group, whereas the water consumption in the apigenin and E2 group was declined to the level of the sham group. Immunohistochemistry of submandibular glands revealed the downregulation of AQP5 expression in xerostomia mice compared to control. Apigenin, or E2 treatment, upregulated AQP5 expression in xerostomia mice. In conclusion, apigenin, a single active component of herbal extract, upregulated AQP5 expression in HSG cells via activation of ERα signaling and restored saliva flow rates in OVX mice. These results revealed apigenin as a single active component of herbal extract with the potential to treat xerostomia.

## Introduction

Xerostomia is the subjective feeling of oral dryness (Delli et al., 2014). The main symptoms of xerostomia are thick saliva, chapped lips, and abnormal taste; it destroys oral functions such as chewing, swallowing, and speaking (Liu et al., 2018). Xerostomia has a significant negative effect on patients’ quality of life (Millsop et al., 2017). Xerostomia affects millions of patients throughout the world, and the prevalence is between 12 and 30% (Tanasiewicz et al., 2016). Reports from the literature suggest that xerostomia affects mostly menopausal women and individuals older than 65 years (Minicucci et al., 2013; D R et al., 2014). Clinical studies have demonstrated that estrogen therapy could effectively alleviate oral dryness by augmenting salivary secretion in menopausal women (Eliasson et al., 2003; Lago et al., 2015). However, long-term estrogen therapy possesses a risk for endometrial and breast cancer (Liang and Shang, 2013; Marjoribanks et al., 2017). Therefore, alternative therapeutic approaches to treat xerostomia are still in high demand.

Shreds of evidence from the literature had shown the therapeutic potential of several herbal extracts to treat xerostomia (Murakami et al., 2009; Chang et al., 2015). However, the cocktail of multiple components in the herbal extract is a key hurdle to unraveling the molecular mechanism and mode of action of the therapy. To overcome this issue, researchers are currently focused on isolating a single active therapeutic component from the herbal extract. Flavonoids, alkaloids, and polysaccharides are commonly isolated active therapeutic components from plant extracts (Almadi and Almohaimede, 2018; Ibrahim et al., 2018). The therapeutic potential and mechanisms of action of these active components, from plant extract, in treating xerostomia are not fully understood.

Aquaporins (AQPs), as transmembrane channel proteins, mediate transcellular water permeability (Delporte et al., 2016). Aquaporin5 (AQP5) is specifically expressed in salivary glands and plays an important role in salivary secretion (Hosoi, 2016). The decreased expression or abnormal distribution of AQP5 diminishes saliva secretion (Krane et al., 2001; Matsuzaki et al., 2012). AQP5 null mice show a 30% reduction in salivary secretion compared to wild type mice (WT) (Ma et al., 1999). Similarly, irradiated rats show decreased saliva secretion and AQP5 protein expression (Li et al., 2006). Since xerostomia is common in postmenopausal women, estrogen deficiency could play a role in the development of this disease. Salivary epithelium expresses a functional estrogen receptor (ER) *α* and ERβ (Tsinti et al., 2009; Wei et al., 2019). The estrogen response element (ERE) is located in the promoter region of the AQP5 gene (Kobayashi et al., 2006; Jiang et al., 2015). This suggests AQP5 as a downstream signaling protein of estrogen signaling. Therefore, the single active component from plant extract that can modulate estrogen AQP5 signaling in the salivary gland could be a possible drug to rescue xerostomia.

In this study, we aimed to identify a single active herbal component as a therapeutic for xerostomia. First, the effect of apigenin (flavonoid), dauricine (alkaloids), protopine (alkaloids), or lentinan (polysaccharides) on AQP5 transcription was screened *in vitro*. We further investigated the mechanism of apigenin-induced AQP5 transcription in human submandibular gland (HSG) cells. The effect of overexpression and knockdown of ERα in HSG cells on AQP5 expression was analyzed. Since only the apigenin upregulated AQP5, docking analysis was performed to unravel the apigenin-specific binding sites in ERα. The effect of apigenin treatment on xerostomia was further investigated in ovariectomized (OVX) mice as a xerostomia model. Our results revealed apigenin as a single active component of herbal extract with the potential to treat xerostomia *via* modulating ERα-AQP5 signaling.

## Material and Methods

### Chemicals and Reagents

HSG cell line was provided by Prof. Hongchen Sun from the Stomatological Hospital of Jilin University. Apigenin and estradiol (E2) (Baoji Herbest Bio-Tech Co., Ltd. Shanxi, China), fetal bovine serum (FBS, Sijiqing Biological Engineering Materials Co. Ltd., Hangzhou, China), Dulbecco’s modified Eagle’s medium (DMEM, Thermo Fisher Scientific, Shanghai, China), EndoFectin Max Transfection Reagent (GeneCopoeia, Rockville, MD, United States), luciferase reported detection reagents (Promega, Madison, WI, United States), total RNA extraction kit (Shanghai, Yeasen, China), anti-mouse monoclonal AQP5 antibodies (Santa Cruz Biotechnology, Dallas, TX, United States), anti-mouse monoclonal ERα antibodies (Santa Cruz Biotechnology, Dallas, TX, United States), anti-rabbit ERα antibodies (Bioss Biosynthesis Biotechnology, Beijing, China), anti-mouse and anti-rabbit secondary antibodies (Bioss Biosynthesis Biotechnology, Beijing, China), and Chromatin Immunoprecipitation (ChIP) Assay Kit (Beyotime, China) were used in this study.

### Cell Culture and Plasmid Transient Transfection

The HSG cells were seeded and cultured in a 10 cm culture dish with DMEM containing 10% FBS, then incubated at 37°C in a humidified incubator supplied with 5% CO2. The AQP5 promoter–luciferase (AQP5p-luc) plasmid was constructed by amplifying the AQP5 promoter sequence, from -2,000 bp to + 200 bp (Homo sapiens), then linking to pGL3 basic vector *via* KpnI and XhoI sites to create an artificial pGL3/AQP5 promoter–reporter system. The ERα plasmid and ERαshRNA plasmid were purchased from Genepharma, Inc.

The HSG cells were seeded into 48-well plates containing 1% FBS, transfected with 100 ng appropriate AQP5p-luc plasmid together with 25 ng pREP7 plasmid, or/and 10 ng ERα/ErαshRNA plasmid using transfection reagent following the manufacturer’s protocol. HSG cells were seeded into 6-well plates containing 1% FBS and transfected with 1 µg ERα/ERαshRNA plasmid using transfection reagent following the manufacturer’s protocol.

### Dual-Luciferase Reporter Assay

HSG cells were seeded in a 48-well plate with a DMEM containing 1% FBS. Cells of each well were transfected with 100 mg appropriate AQP5p-luc plasmid together with 25 ng pREP7 plasmid for 48 h. Then the cells were stimulated by dauricine (1 μM), protopine (1 μM), lentinan (1 μM), apigenin (1 μM), or E2 (0.1 μM) for 24 h. The apigenin was divided into 0.01, 0.1, and 1 μM. The cells were lysed in 65 μL passive lysis buffer rocking on ice for 30 min. The lysate was transferred into a new tube and centrifuged at a speed of 13,500 rpm for 10 min. For firefly luciferase activity detection, 30 μL of the supernatant was measured by using the dual-luciferase reporter assay system. The ratio of firefly luciferase activity to Renilla luciferase activity was calculated as relative luciferase activity.

### Real-Time Quantitative PCR

HSG cells (Jaiboonma et al., 2020) were seeded in 6-well plates with a DMEM containing 1% FBS. The cells were stimulated with apigenin (0.01, 0.1, and 1 μM) and E2 (0.1 μM) for 48 h. The total RNA from differently treated cells was extracted using a total RNA extraction reagent. RNA was then reverse-transcribed by first strand cDNA synthesis supermix for qPCR and amplified using SYBR Green Master Mix by the real-time PCR detection system. Primers used for qPCR were as follows: human AQP5 (forward primer: 5′-TGC​CAT​CCT​TTA​CTT​CTA​CCT​G-3′, reverse primer: 5′-CTC​ATA​CGT​GCC​TTT​GAT​GAT​G-3′) and human β-actin (forward primer: 5′-GGC​ACC​ACA​CCT​TCT​ACA​ATG​AGC-3′, reverse primer: 5′-GAT​AGC​ACA​GCC​TGG​ATA​GCA​ACG-3′). The thermal cycling condition for PCR amplification was 95°C for 5 min, 40 cycles of 95°C for 10 s, 60°C for 30 s, followed by 40°C for 20 min. The relative expression ratio was calculated from real-time PCR efficiencies and the crossing point deviation of a given gene vs. *β*-actin house-keeping gene. In each independent experiment, the mean gene expression ratios obtained with regularly submerged cultures were given a value of 1 (fold).

### Western Blot Assay

HSG cells were seeded in a 6-well plate with a DMEM containing 1% FBS. Cells of each well were stimulated with apigenin (0.01, 0.1, and 1 μM) and E2 (0.1 μM) for 48 h. To verify whether apigenin upregulated AQP5 transcription through the ERα pathway HSG cells were seeded in 6-well plates containing 1% FBS, transfected with 1 µg ERα/ERαshRNA plasmid using transfection reagent for 48 h, and induced with 1 μM apigenin for 24 h. The cells were lysed on ice with RIPA for 30 min. The submandibular glands tissues of mice were lysed on ice with RIPA. The protein samples were resolved on 12% SDS-PAGE and transferred to the PVDF membrane. The membranes were incubated with 5% (w/v) skimmed milk in TBST, followed by incubation with primary antibodies (AQP5 1:200, *β*-actin 1:1,000) at 4°C overnight. The membranes were further incubated with HRP conjugated respective goat anti-mouse (1:500) secondary antibody IgG at room temperature for 1 h. Signals were detected using ECL plus chemiluminescence kit on X-ray film. The protein expression in the Western blots was quantified using Image J software.

### Immunofluorescence Study

HSG cells were seeded in DMEM complemented with 1% FBS. The cells were stimulated with 1 μM apigenin and 0.1 μM E2 for 48 h. The cells were fixed with 4% paraformaldehyde for 10 min, permeabilized in 0.2% Triton X-100 for 10 min, and blocked with 1% bovine serum albumin (BSA) for 1 h. Then the cells were incubated with anti-mouse AQP5 antibodies in 1:100 dilution at 4°C overnight, followed by incubation with fluorophore-conjugated secondary antibodies 1:500 for 1 h at room temperature. DAPI staining for 5 min was carried out after secondary antibody incubation.

To verify whether apigenin upregulated AQP5 transcription through the ERα pathway, HSG cells were transfected with ERα/ERαshRNA plasmid using transfection reagent for 48 h and induced with 1 μM apigenin for 24 h. The cells were fixed with 4% paraformaldehyde for 10 min, permeabilized in 0.2% Triton X-100 for 10 min, and blocked with 1% bovine serum albumin (BSA) for 1 h. Then the cells were incubated with anti-mouse AQP5 antibodies in 1:100 dilution at 4°C overnight and anti-rabbit ERα antibodies in 1:100 dilution for 2 h at room temperature. The cells were incubated with fluorophore-conjugated goat anti-mouse (red) secondary antibodies 1:500 for 1 h at room temperature, followed by fluorophore-conjugated goat anti-rabbit (green) secondary antibodies 1:1,000 for 1 h at room temperature. DAPI staining for 5 min was carried out after secondary antibody incubation. Staining was detected using fluorescent microscopy (model IX71; Olympus, Tokyo, Japan).

### ChIP Assay

HSG cells were treated with 1 μM apigenin for 48 h. The chromatin immunoprecipitation (ChIP) assays were performed according to the manufacturer’s protocol (Beyotime Co.). Chromatin solutions were sonicated and incubated with anti-ERα and rotated overnight at 4°C. DNA–protein cross-links were reversed and chromatin DNA was purified and subjected to PCR analysis. The primers ERE1 (forward primer: 5′-GGA​ACT​GGA​AGA​AAG​TGT​CA-3′, reverse primer: 5′-TGC​CTT​TTG​CTG​TCT​TAG​TC-3′), ERE2 (forward primer: 5′- TTG​GGA​GGT​CAG​TGG​TGC-3′, reverse primer: 5′-TGG​AAG​GCT​GGC​GTT​TT-3′), ERE3 (forward primer: 5′-CAA​AAC​GCC​AGC​CTT​CCA​A-3′, reverse primer: 5′-TCC​TCC​TTT​TCC​TCC​TGC​GAC -3′), ERE4 (forward primer: 5′-AGC​TAG​ACG​CCC​CGA​GGT​CG-3′, reverse primer: 5′-TCT​CCG​TCG​TCC​AGC​GCA​AC-3′), which were designed to amplify the AQP5 promoter region that contains ERα binding sites by Jaspar database, were used. After amplification, PCR products were resolved on a 1.5% agarose gel.

### Autodock Analyses

For docking purposes, the structure of apigenin (PubChem CID: 5280443), E2 (PubChem CID: 5756), and lentinan (PubChem CID: 37723) were retrieved from the PubChem Compound repository of small molecules in NCBI. The macromolecular structures of the human ERα ligand binding region were retrieved from the PDB protein data bank. Docking was carried out by AutoDock software to analyze the binding energy and amino acid residues of apigenin and ERα in the docking model.

### Animal Study

Twenty-four female ICR mice (aged 6 weeks, weighing 30 ± 2 g) were purchased from the Hua Fukang Biotechnology Co., Ltd. (Beijing, China). All mice were free to obtain soy-based food and water under the light/dark cycle for 12/12 h at a constant temperature of 25 ± 1°C. The mice were divided randomly into four groups (sham operation, OVX/control, OVX/apigenin, and OVX/E2) with six mice in each group. For ovariectomy and sham surgery, the mice were anesthetized with intraperitoneal administration of 10 mg/kg 0.7% sodium pentobarbital. An incision was made in the middle of the abdomen and then the bilateral ovaries were removed. In the sham group, the ovaries were exposed and a small piece of adipose tissue was removed. The incision was layered and sutured. Then the mice received daily drug treatment by oral gavage. The OVX/apigenin group mice were gavaged with apigenin (dissolved in 0.5% carboxymethyl cellulose sodium) 50 mg/kg/day (Yang et al., 2018). The OVX/E2 group mice were gavaged with E2 (dissolved in 0.5% carboxymethyl cellulose sodium) 1 mg/kg/day (Liu et al., 2018). The sham operation group and OVX/control group mice were administrated by 0.5% carboxymethyl cellulose sodium in the same way. The saliva secretion and water consumption were recorded before ovariectomy and after 4, 8, and 12 weeks of drug administration as described previously (Lai et al., 2016; Liu et al., 2018). After 12 weeks of drug administration, mice were anesthetized and submandibular glands were used for immunohistochemistry and Western blot assay.

### Saliva Secretion and Water Consumption Assessment

The saliva secretion was measured by a previously described method before ovariectomy and after 4, 8, and 12 weeks of drug administration (Nakamura et al., 2004; Tajiri et al., 2019). Before the saliva was collected, the mice were fasted for the night. The mice were routinely anesthetized with intraperitoneal administration of 10 mg/kg 0.7% sodium pentobarbital. Then pilocarpine (1.0 mg/kg) was injected subcutaneously. We collected saliva from the oral cavity using pre-weighed pieces of cotton every 5 min for a total of 30 min, then weighed the pieces of cotton again. The saliva secretion index was calculated by the amount of increase in weight (mg)/body weight (g).

The water consumption for 7 days was recorded before ovariectomy and after, 4, 8, and 12 weeks of drug administration. The average water consumption index of each group was calculated by the water consumption (ml)/body weight (g), and then the changes among each group were observed.

### Histology and Immunostaining

After 12 weeks of drug administration, mice were anesthetized and submandibular glands were removed by routine surgery and fixed in 4% polyformaldehyde. Then the specimen was dehydrated with alcohol gradient, transparent to xylene, embedded in paraffin, and cut into 5-μm-thick tissue sections. For immunohistochemistry, tissue sections were incubated with primary antibodies, AQP5 (1:100) at 4°C overnight. Then the tissue sections were incubated with biotinylated goat anti-rabbit/mouse IgG antibody at room temperature for 60 min and followed by DAB color rendering hematoxylin counterstaining for 3 min. Then the same tissue sections were counterstained with hematoxylin and eosin (H&E) staining. Staining was visualized in the same structural area and quantified using a model IX71 light microscope (Olympus, Tokyo, Japan). Plaque number and staining area were calculated by Image J software.

### Statistical Analysis

All data are presented as mean ± standard deviation (SD). Statistical significance was evaluated using a one-way analysis of variance. Each experiment was repeated at least three times. Statistical analysis was performed using SPSS 22.0 statistics software. Image J software was used for data analysis in Western blot and immunohistochemical staining. Differences were considered to be statistically significant at *p* < 0.05.

## Results

### Apigenin, a Natural Flavone, Screened to Activate AQP5 Transcription

**FIGURE 1:**
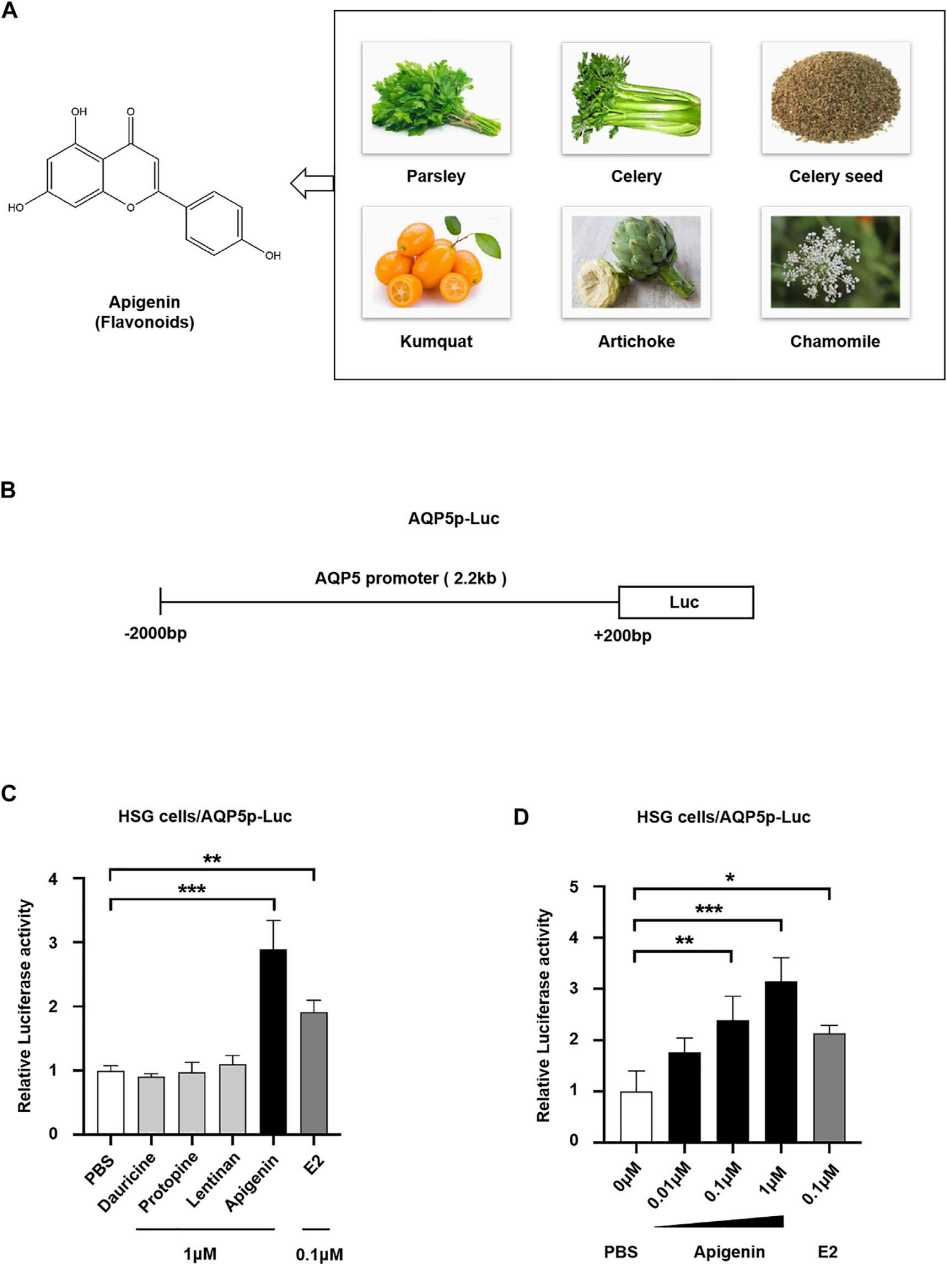
Natural product apigenin upregulates AQP5 transcription. **(A)** Structure of apigenin. **(B)** Luciferase reporter pGL3/AQP5 promoter (AQP5p) plasmid. **(C)** Relative luciferase activity showing the AQP5p activation induced by apigenin in HSG cells. **(D)** Apigenin activated the AQP5p in dose dependence. Data are presented as the mean ± SD, *n* = 3. Significant effect of the treatment, *p < 0.05, **p < 0.01.

To screen the natural products activating AQP5 transcription, we performed a dual-luciferase reporter assay. In this study, three categories of effective components from plant extract, that is, flavonoids (apigenin), alkaloids (dauricine and protopine), and polysaccharides (lentinan) were selected to screen their effect on AQP5 expression. Apigenin, a natural flavone, can be extracted from parsley, celery, chamomile, and so on (Figure 1A). Estradiol (E2), a positive transcriptional regulator of AQP5, was used as a positive control. We transfected the luciferase reporter pGL3/AQP5p (-2,000-+200 bp, *Homo sapiens*) plasmid (Figure 1B) into HSG cells, induced by PBS, 1 μM dauricine, protopine, lentinan, apigenin, and 0.1 μM E2, and then tested AQP5 transcription. The data showed that dauricine, protopine, and lentinan did not affect the AQP5 transcription, whereas E2 treatment enhanced AQP5 transcription by approximately 2.0-fold. Interestingly, apigenin (1 μM) robustly induced AQP5 transcription by 3.0-fold (Figure 1C). Furthermore, to investigate the dose-dependent effect of apigenin on the AQP5 transcription, we performed a dual-luciferase reporter assay in HSG cells in the presence of different concentrations of apigenin. Apigenin upregulated AQP5 transcription in a dose-dependent manner (Figure 1D). The data indicate that apigenin, a natural flavone, activates AQP5 transcription.

### Apigenin Upregulates AQP5 Transcription and Expression

**FIGURE 2:**
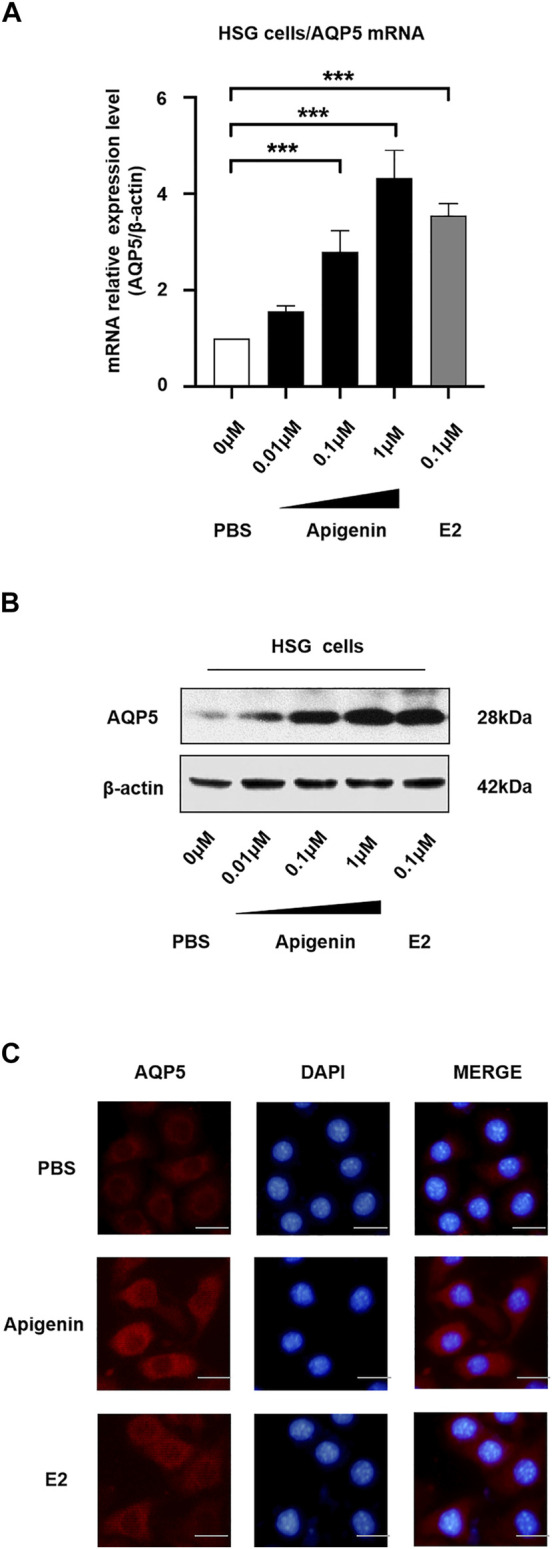
Apigenin upregulates AQP5 transcription and expression. **(A)** mRNA relative expression level (AQP5/β-actin) showing the AQP5 transcription in HSG cells by qPCR. **(B)** Apigenin upregulates AQP5 expression. Representative Western blot image showing endogenous AQP5 protein expression in HSG cells. **(C)** Immunofluorescence staining showing the AQP5 protein level and localization in HSG cells. Bar scale, 50 μm. Data are presented as the mean ± SD, *n* = 3. Significant effect of the treatment, *p < 0.05, **p < 0.01.

To monitor the changes of the endogenous AQP5 transcription level, we performed the real-time quantitative PCR in HSG cells induced by different concentrations of apigenin. Apigenin upregulated AQP5 transcription in a dose-dependent manner, and the AQP5 mRNA relative expression level was increased by 4.0-fold compared to the control group (Figure 2A). The effect of apigenin (1 μM) on AQP5 transcription was more prominent than that of E2 (0.1 μM). These data suggest the AQP5 transcription potential of apigenin.

To verify the endogenous AQP5 protein level and localization in HSG cells, we performed Western blot and immunofluorescence staining. We observed there was a dose-dependent increase in AQP5 protein level in the HSG cells treated with apigenin (Figure 2B). The results of Western blot analysis were in accordance with the results of qPCR. As shown in Figure 2C, AQP5 protein expression was upregulated in the apigenin-treated group. The AQP5 protein was mainly localized in the cell membrane and cytoplasm. Taken together, these data indicate that apigenin upregulates AQP5 transcription and expression in HSG cells.

### Apigenin Upregulates Transcription and Translation of AQP5 Through the ERα Pathway

We demonstrated that apigenin upregulates AQP5 transcription. Apigenin is a flavone with estrogen activity; therefore, we analyzed potential ERα binding sites in the AQP5 promoter and explored whether the ERα pathway involved in the AQP5 transcriptional activation.

**FIGURE 3:**
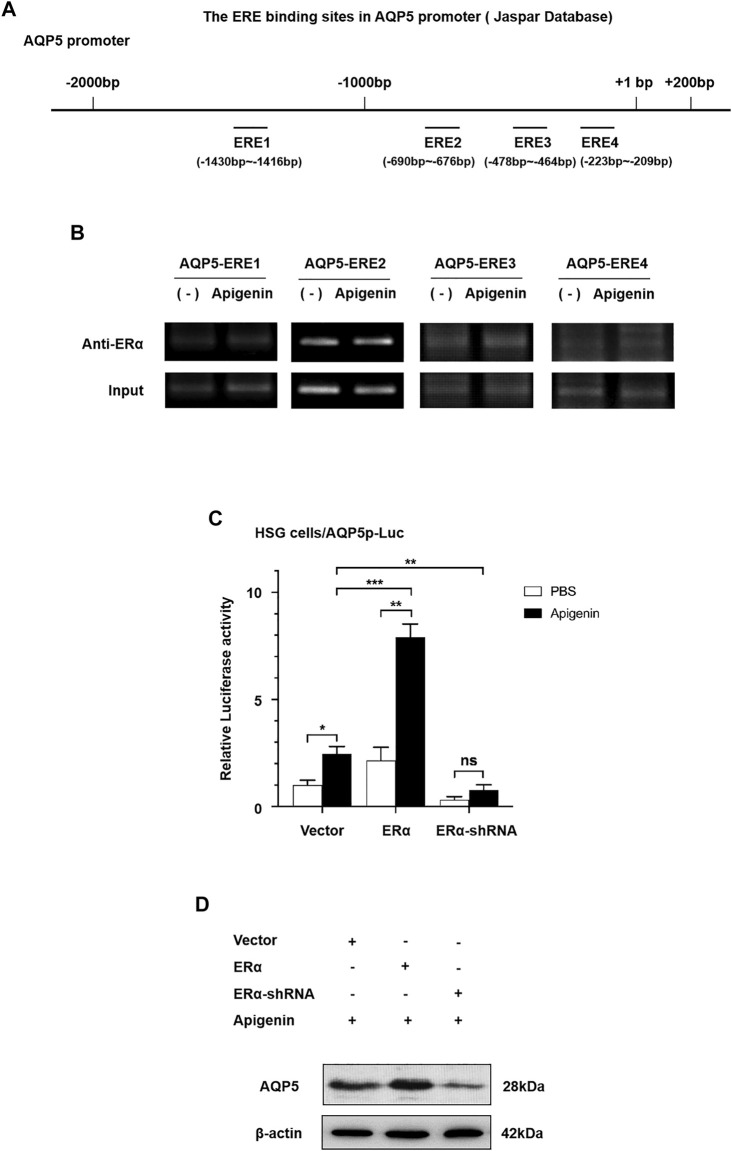
Apigenin upregulates the transcription and translation of AQP5 through the ERα pathway. **(A)** Scheme showing AQP5 promoter sequence with ERα binding sites. **(B)** ChIP results showed that ERα occupancy was apparently increased at ERE3 in the presence of apigenin. **(C)** Relative luciferase activity (the indication of AQP5 expression) in ERα-overexpressed and -knocked down with or without 1 μM apigenin in HSG cells. **(D)** Western blot analysis showing the effect of apigenin treatment on AQP5 protein expression in ERα-overexpressed and -knocked down HSG cells. Data are presented as the mean ± SD, *n* = 3. Significant effect of the treatment, *p < 0.05, **p < 0.01.

First, to evaluate the existence of ERα binding sites in the AQP5 promoter sequence, we searched the AQP5 promoter in NCBI databases and predicted transcription factor binding sites in the JASPAR database. As shown in Figure 3A, four ERα binding sites are located in the AQP5 promoter sequence. The ERE sequence is at −1,430 to −1,416, −690 to −676, −478 to −464, and −223 to −209 bp in AQP5 promote. These data suggest that ERα binding sites exist in the AQP5 promoter sequence.

To ascertain the direct recruitment of the ERα complex on the AQP5 promoter, ChIP assays were performed with ERα antibodies in HSG cells. In order to further verify ERE binding sites in apigenin and AQP5 promoter sequences, we designed 4 pairs of ChIP primers, whose products were −1,444 to −1,211, −883 to −643, −660 to −442, and −374 to −143 bp, respectively, included ERE binding sites predicted by JASPER database. ChIP results showed that ERα occupancy was apparently increased at ERE3 in the presence of apigenin (Figure 3B). These data suggest the direct increased recruitment of ERα on the AQP5 promoter is the response to apigenin.

Then, we aimed to verify whether apigenin upregulated AQP5 transcription through the ERα pathway. We performed a dual-luciferase reporter assay and Western blot by gene overexpression and interference of ERα. As shown in Figure 3C, the overexpression of ERα induced AQP5 activation in HSG cells by 2.0-fold. Intriguingly, while interference of ERα, the AQP5 activation was downregulated. Meanwhile, an approximately 4.0-fold induction of AQP5 activation was observed in apigenin-treated ERα overexpressed HSG cells. This effect was nullified in ERα-knocked down HSG cells. A similar effect of apigenin in AQP5 expression was observed in ERα-overexpressed and -knocked down in Western blot in HSG cells (Figure 3D). Taken together, the aforementioned results indicate that apigenin activates AQP5 transcription and upregulates the AQP5 protein expression through the ERα pathway.

### Apigenin Specifically Binds to ERα Within Its Ligand Binding Domain by Molecular Docking of Apigenin to ERα

**FIGURE 4:**
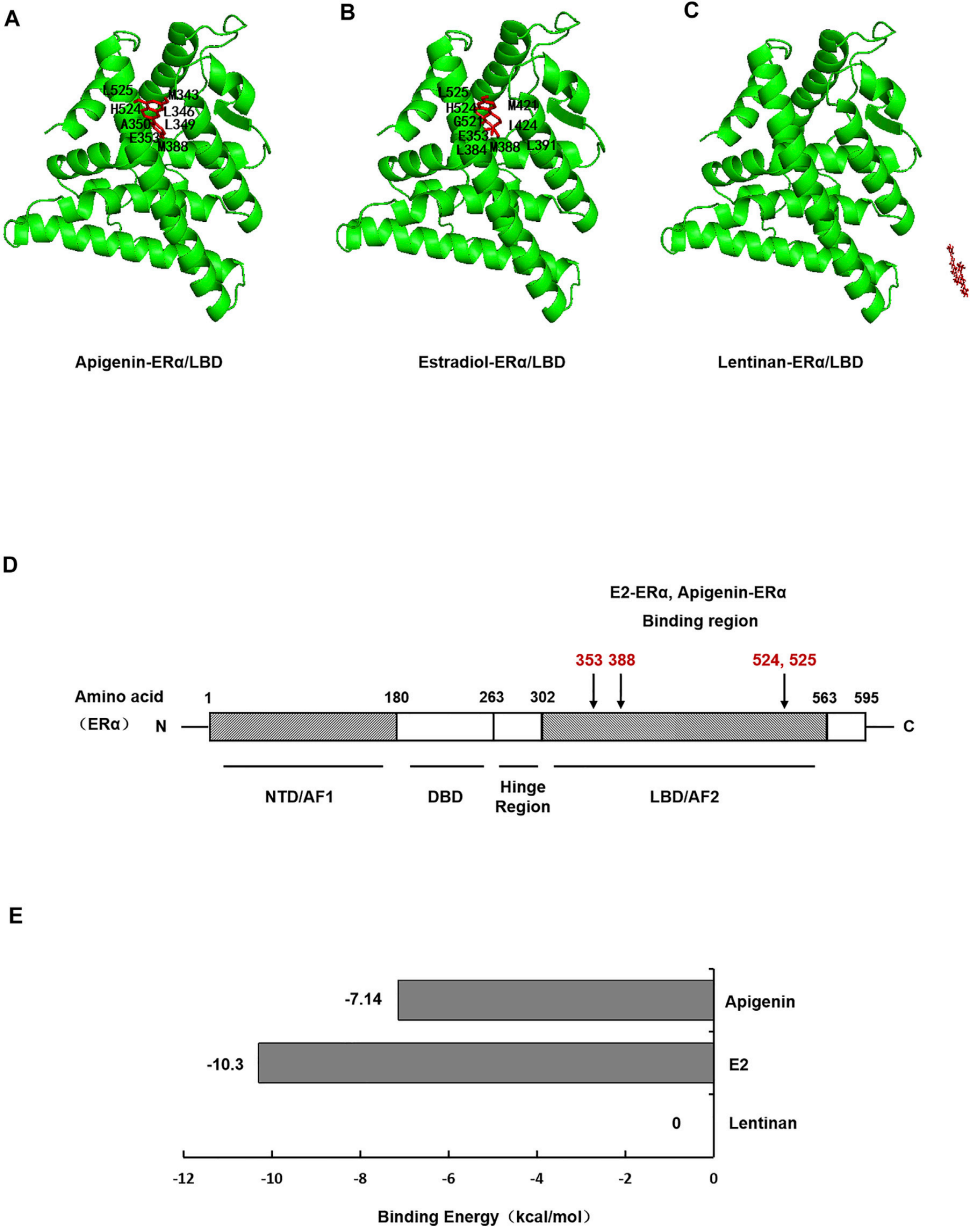
Molecular docking shows specific binding of apigenin to ERα within the E2-ligand binding domain. Images showing the binding site of apigenin **(A)**, E2 **(B)**, and lentinan **(C)** in ERα. **(D)** Interaction of amino acid residues located in the ligand binding domain (LBD) of ERα. **(E)** Binding energy of apigenin, E2, and lentinan with ERα.

To investigate whether apigenin functioned as a ligand of ERα protein, then formed complexes with ERα protein, autodock software was performed to dock the molecule of apigenin to ERα protein. E2, the natural ligand of ERα, was set as a positive control, while lentinan was a negative control. First, the docking results showed that apigenin is located within the ligand binding (LBD) site of ERα (Figure 4A), at the same position in ERα as its natural ligand E2 (Figure 4B). The negative control lentinan could not enter the LBD region of ERα (Figure 4C).

ERα protein contains 595 amino acids, which is composed of three functional domains: the NH2-terminal domain (NTD/AF1, 1–180 amino acids), the DNA binding domain (DBD, 180–263 amino acids), and the COOH-terminal ligand binding domain (LBD/AF2, 302–563 amino acids) (Jia et al., 2015). In our docking study, apigenin was found to interact with the LBD of ERα with the amino acid residues MET343, LEU346, LEU349, LAA350, GLU353, MET388, HIS524, and LEU52. The positive control E2 was found to interact with LBD of ERα with the amino acid residues GLU353, LEU384, MET388, LEU391, ARG394, MET421, ILE424, GLY521, HIS524, and LEU525 (Table 1). Interestingly, the amino acid residues of apigenin binding to ERα were similar to E2 with the same amino acid residues including GLU353, MET388, HIS524, and LEU525 (Figure 4D), whereas the negative control lentinan was free outside of the LBD of ERα. The results suggest that apigenin is a natural product that plays an estrogen-like role.

**TABLE 1:** Amino acid residues in the apigenin, E2, and lentinan binding sites of ERα.

Ligand	Macromolecule	Amino acid residues
Apigenin	ERα	MET343, LEU346, LEU349, LAA350, GLU353, MET388, HIS524, LEU525
E2	ERα	GLU353, LEU384, MET388, LEU391, ARG394, MET421, ILE424, GLY521, HIS524, LEU525
Lentinan	ERα	—

Finally, the binding energy (Figure 4E) between apigenin and ERα was −7.14 kcal/mol, which approached the binding energy between E2 and ERα. However, the binding energy between lentinan and ERα was 0 kcal/mol. These data indicate that apigenin has strong binding abilities to ERα. Taken together, the aforementioned results suggest that apigenin specifically bound to ERα within its ligand binding domain and then activated the AQP5 transcription.

### Apigenin Ameliorates the Impairment of Saliva Secretion and Reduced the Water Consumption Rate in OVX Mice

**FIGURE 5:**
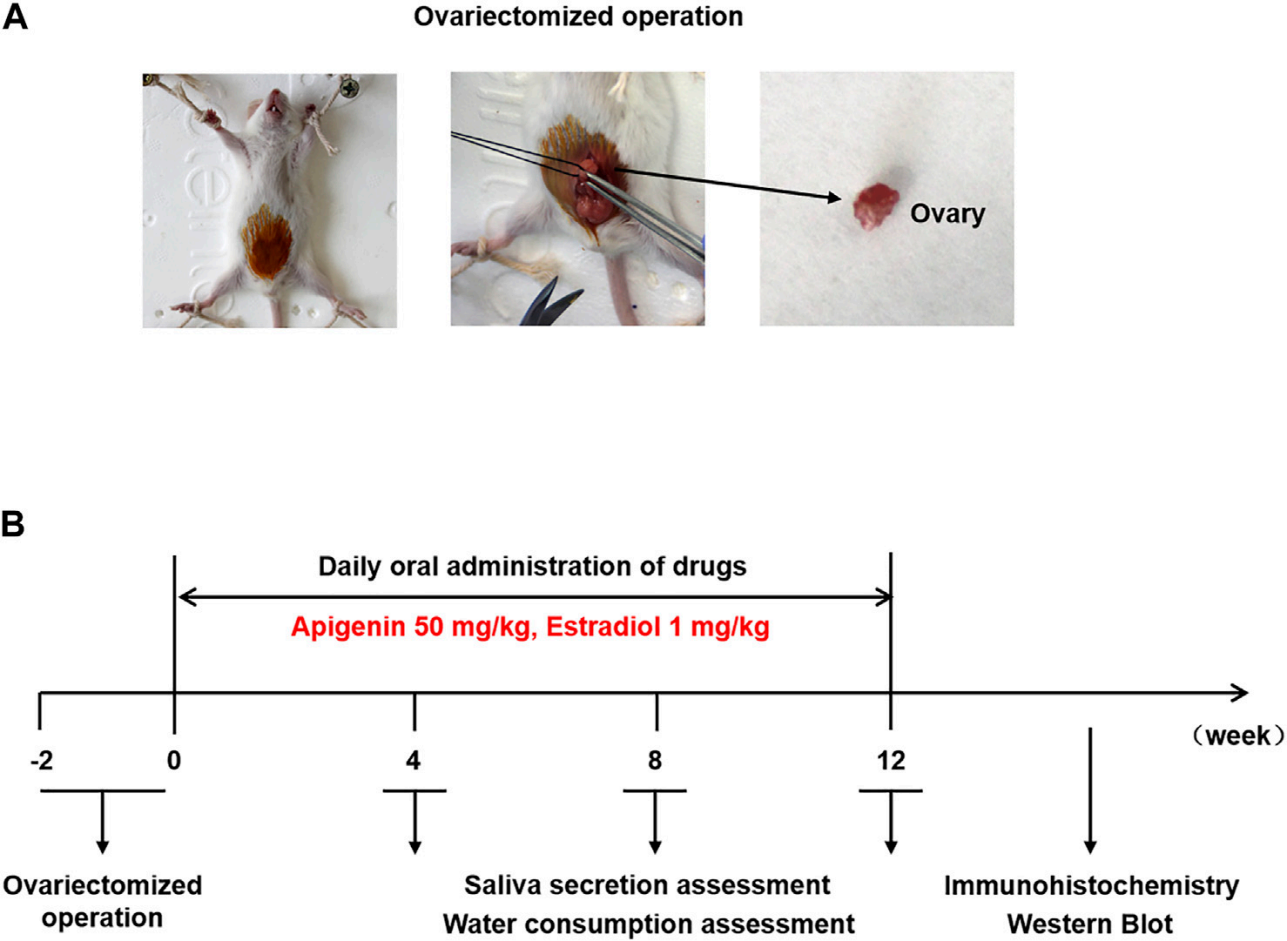
**(A)** Ovariectomized operation. **(B)** Scheme of animal experiment and treatment.

To verify the effect of apigenin *in vivo*, we constructed the OVX mice model. The mice were divided randomly into four groups, including sham operation, OVX/control, OVX/apigenin (drug group), and OVX/E2 (positive group) with six mice in each group. The animal study was performed as illustrated in Figure 5.

**FIGURE 6:**
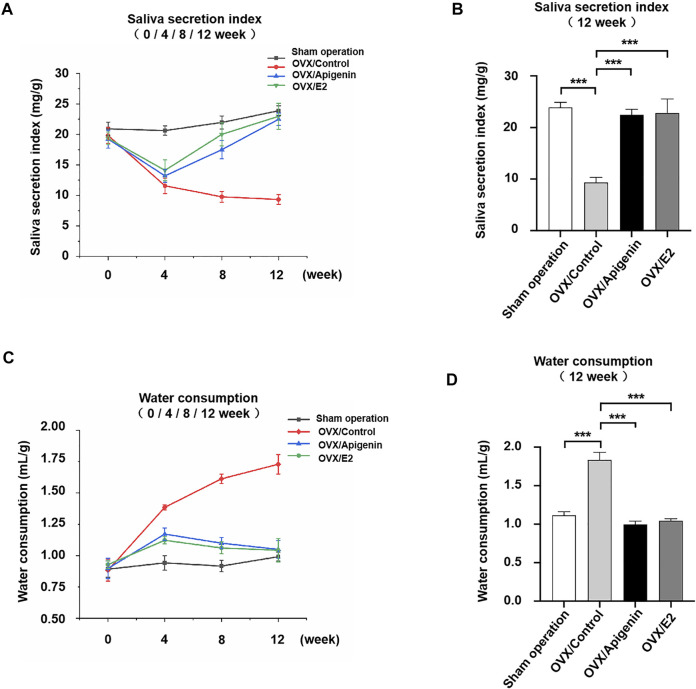
Apigenin ameliorates the impairment of saliva secretion and reduced the water consumption rate in OVX mice. **(A)** Salivary secretion index before ovariectomy and after 4, 8, and 12 weeks of treatments. **(B)** Salivary secretion index after 12 weeks of treatment. **(C)** Water consumption rate before ovariectomy and after 4, 8, and 12 weeks of treatments. **(D)** Water consumption rate after 12 weeks of treatment. Data are presented as the mean ± SD, *n* = 6. Significant effect of the treatment, *p < 0.05, **p < 0.01.

The saliva secretion was measured before ovariectomy and after 4, 8, and 12 weeks of treatment in Figure 6A. Saliva secretion was similar in the four groups before ovariectomy. The saliva secretion in the sham operation group did not change significantly at various stages, and the salivary secretion index was 20–25 mg/g. The saliva secretion in the OVX/control group decreased sharply at 4 weeks after administration, which was reduced to half of that in the sham operation group. The saliva secretion decreased gradually at 8–12 weeks after administration and was maintained at a low-level stage. At 12 weeks, the saliva secretion of the OVX/control group was reduced to 1/3 of that of the sham operation group. The saliva secretion in the OVX/apigenin group and OVX/E2 group was significantly lower at 4 weeks than that of the sham operation group. However, after 4 weeks of administration, the saliva secretion index began to increase, and the saliva secretion index increased gradually and approached the level of the sham operation group at 4–12 weeks of administration. At 12 weeks of administration, the saliva secretion index of the OVX/apigenin group and OVX/E2 group almost reached the level of the sham operation group. These results indicate that apigenin can significantly ameliorate saliva secretion in OVX mice, which is effective from 4 to 12 weeks.

To further illustrate the results, we analyzed the saliva secretion index in each group after 12 weeks of administration. As shown in Figure 6B, the saliva secretion index in the OVX/control group was 1/3 of that in the sham operation group. However, the saliva secretion index of the OVX/apigenin group and OVX/E2 group was completely different from that of the OVX/control group, while close to that of the sham operation group. These results indicate that apigenin rescues the impairment of saliva secretion in OVX mice.

To demonstrate the effects of apigenin on the thirsty symptom, we measured the water consumption rate before ovariectomy and after 4, 8, and 12 weeks of treatment in Figure 6C. The water consumption in the sham operation group showed no significant change at each stage, and the water consumption index was 0.75–1.0 ml/g. The water consumption in the OVX/control group increased to nearly 1.5 times of the sham operation group at 4 weeks of administration. During 8–12 weeks of administration, water consumption increased slowly and remained at a high level. At 12 weeks, the water consumption in OVX/control group increased nearly 1.7 times to that of the sham operation group. These results indicate that water consumption in ovariectomized mice increased significantly, suggesting the symptoms of xerostomia. Water consumption in the OVX/apigenin group and OVX/E2 group was higher at 4 weeks than that in the sham operation group. However, after 4 weeks of administration, water consumption began to decline, decreased gradually, and was close to the level of the sham operation group. At 12 weeks of administration, the water consumption of the OVX/apigenin group and OVX/E2 group almost reached the level of the sham operation group. These results indicate that apigenin significantly reduces water consumption in ovariectomized mice, which is effective at 4–12 weeks.

We further analyzed water consumption in each group after 12 weeks of administration. As shown in Figure 6D, the water consumption in the OVX/control group was about 1.7 times that in the sham operation group. The water consumption in the OVX/apigenin group and OVX/E2 group was close to that in the sham operation group. The results suggest that apigenin relieves the thirsty symptom in OVX mice.

### Apigenin Upregulates the Expression of AQP5 in the Submandibular Glands of OVX Mice

**FIGURE 7:**
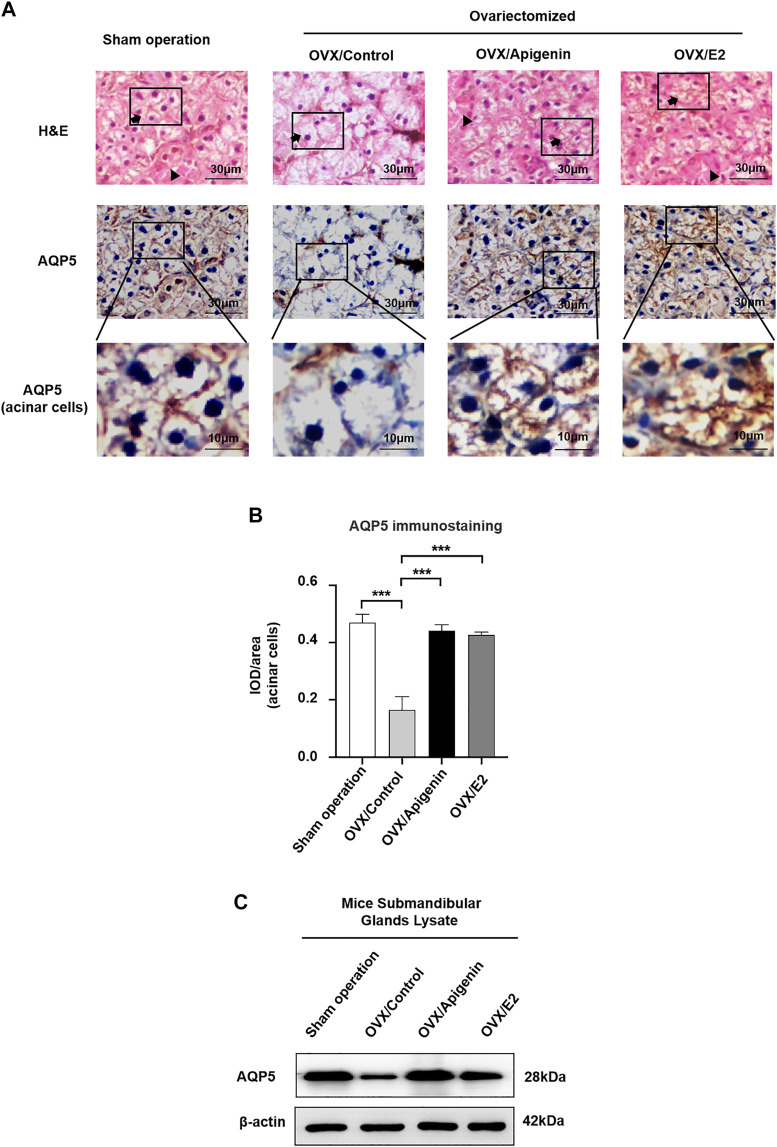
Apigenin treatment upregulates AQP5 expression in the submandibular gland of OVX mice. **(A)** Histological images (H&E stained) and immunohistochemistry images of AQP5 of mice submandibular gland tissues. Arrows, acinar cells. Triangles, ducts cells. **(B)** Quantitative analysis of immunohistochemistry images. **(C)** AQP5 protein expression in the submandibular gland lysate. Data are presented as the mean ± SD, *n* = 6 mice per group. Significant effect of the treatment, *p < 0.05, **p < 0.01.

Based on the fact that apigenin ameliorates the impairment of saliva secretion in the OVX mice, we next investigated the expression of the key protein AQP5 that is involved in saliva secretion in the submandibular glands. As indicated by H&E staining, there was no significant change in the histology of submandibular gland tissue among the groups (Figure 7A).

As described previously (D’Agostino et al., 2020), both the apical and basolateral membranes of acinar cells display positive AQP5 labeling in mice (Delporte, 2014; Hosoi et al., 2020). In addition, intercalated ducts express AQP5 (Larsen et al., 2011). As shown in Figure 7A, in the sham operation group, AQP5 immunostaining was distributed at the membrane of acinar cells. In the OVX/control group, AQP5 immunostaining at the membrane of acinar cells, especially at the apical membrane, was severely declined. While AQP5 immunostaining at the membrane of acinar cells was augmented approaching the level of the sham operation group in the OVX/apigenin and OVX/E2 group. In quantitative analysis (Figure 7B), the AQP5 immunostaining in acinar cells in the OVX/control group declined severely compared to the sham operation group. As indicated, the AQP5 immunostaining in the OVX/apigenin and OVX/E2 groups was augmented approaching the level of the sham operation group. Furthermore, to detect the AQP5 protein expression level in the mice’s submandibular glands, we performed a Western blot analysis. In the OVX/control group, the AQP5 protein level was declined severely compared to the sham operation group. As indicated, the AQP5 protein level in the OVX/apigenin and OVX/E2 groups was augmented approaching the level of the sham operation group (Figure 7C). These data indicate that apigenin upregulates the expression of AQP5 in the submandibular glands in OVX mice.

## Discussion

Xerostomia has high incidence with age, which was demonstrated by the reports from the literature that one in ten for younger and one in four for elder Australians (55 years old as cutoff) experienced dry mouth, with a prevalence of 9.3% among 15- to 34-year-olds, 11% among 35- to 54-year-olds, 17.6% among 55- to 74-year-olds, and 26.5% among those older than 75 years old (Jamieson and Thomson, 2020). Another study also reported xerostomia was one in five (19.1%) by 293 elderly people older than 60 years in Vanini, Brazil (Fornari et al., 2021). Moreover, xerostomia affects more than one-third of postmenopausal women (Nederfors, 2000). Salivary glands are sensitive to changes in female sex steroid blood levels (Agha-Hosseini et al., 2009). The decrease in estrogen levels during menopause is thought to affect salivary secretion (Forabosco et al., 1992). AQP5 is a key protein involved in salivary secretion and its expression downregulates in xerostomia. Effective therapy for xerostomia is still lacking. In this study, we unraveled that apigenin, an active flavone, directly binds with ERα and enhances AQP5 expression and activity in HSG cells via ERα signaling. Furthermore, apigenin treatment alleviated xerostomia and upregulated AQP5 expression in the submandibular gland in OVX mice. The results of this study unravel the therapeutic potential of apigenin for xerostomia and the underlying mechanism.

Apigenin, a natural flavone, is present in a significant amount as a glycosylated form in vegetables (parsley, celery, and onions), fruits (oranges), herbs (chamomile, thyme, oregano, and basil), and plant-based beverages (tea, beer, and wine) (Hostetler et al., 2017; Salehi et al., 2019). Apigenin has antidiabetic and anticancer potential (Yan et al., 2017; Ginwala et al., 2019). Moreover, apigenin treatment had shown a beneficial role in amnesia, Alzheimer’s disease, depression, and insomnia (Venigalla et al., 2015; Salehi et al., 2019). Shreds of literature had reported anti-inflammatory and antimicrobial properties of apigenin, suggesting its application in various diseases. Apigenin is considered safe even in high doses and can be extracted easily from its natural sources. Considering these facts, we tested the effect of apigenin treatment on xerostomia and signaling molecules involved in xerostomia. AQP5 are key proteins involved in the pathophysiology of xerostomia. We found upregulation of ERα/AQP5 protein expression in apigenin-treated HSG cells and the submandibular gland of OVX mice. These findings indicate that apigenin has the potential to activate ERα/AQP5 signaling on salivary glands.

Postmenopausal estrogen deficiency is linked to various diseases, including xerostomia, osteoporosis, diabetes, and other inflammatory diseases (Caputo and Costa, 2014; Baer and Walitt, 2018). Hormone replacement therapy (HRT) had been reported to increase salivary secretion in postmenopausal women (Yalçin et al., 2005; Lago et al., 2015). However, HRT possesses a risk of adverse side effects, including the development of endometrial and breast cancer (Pietrzak et al., 2015). Phytoestrogens are plant-derived dietary compounds with structural similarity to E2 (Rietjens et al., 2017). This structural similarity to E2 enables phytoestrogens to cause estrogenic effects by binding to the estrogen receptors. The docking study revealed the similar binding sites of apigenin and E2 on ERα, indicating apigenin as a decoy of E2. Goto et al. reported that apigenin treatment prevents estrogen deficiency-induced bone loss in OVX mice (Goto et al., 2015). Therefore, apigenin could have the potential to alleviate estrogen deficiency-related diseases including xerostomia.

Reports from the literature and our previous studies revealed the role of AQP5 on the pathophysiology of xerostomia (Matsuzaki et al., 2012). In this study, treatment of apigenin robustly upregulated ERα and AQP5 expression in HSG cells *in vitro* and the submandibular gland of OVX mice. AQP5 expression and activity were increased in apigenin-treated HSG cells with the overexpression of ERα. Interestingly, apigenin failed to increase the AQP5 expression and activity in ERα-knocked down HSG cells indicating the ERα-mediated effect of apigenin on AQP5 expression and activity. Reports from the literature also reported the stimulatory effect of E2 on AQP5 expression (Jiang et al., 2015; Wei et al., 2019). Moreover, the apigenin and E2 treatment increased the salivary secretion index and decreased water consumption in OVX mice in a similar extent. This is the first study to report the anabolic role of apigenin on AQP5 expression and activity via binding with ERα. These results unravel the therapeutic potential of apigenin for xerostomia via ERα/AQP5 signaling. Our results indicate promising potential of apigenin to treat xerostomia.

## Conclusion

Xerostomia is defined as decreased salivary flow or hypofunction of the salivary glands. Among the 4 plant extracts tested in this study, only apigenin showed robust potential to activate the expression. Since AQP5 is a key protein involved in salivary secretion *via* ERα signaling, we further investigated the role of apigenin on ERα/AQP5 pathway-mediated salivary secretion. Apigenin, an active flavone component of herbal extract, upregulated AQP5 expression *via* activation of ERα signaling and restored saliva flow rates in OVX mice. Our results revealed the possible therapeutic potential of apigenin to alleviate the clinical symptoms of xerostomia.

## Data Availability

The original contributions presented in the study are included in the article/Supplementary Material, further inquiries can be directed to the corresponding authors.
