# Supplementary figures and images for: Apigenin, a Single Active Component of Herbal Extract, Alleviates Xerostomia via ERα-Mediated Upregulation of AQP5 Activation
Source: Front Pharmacol. 2022 Feb 21;13:818116. doi: 10.3389/fphar.2022.818116 (PMC8899471; doi:10.3389/fphar.2022.818116)

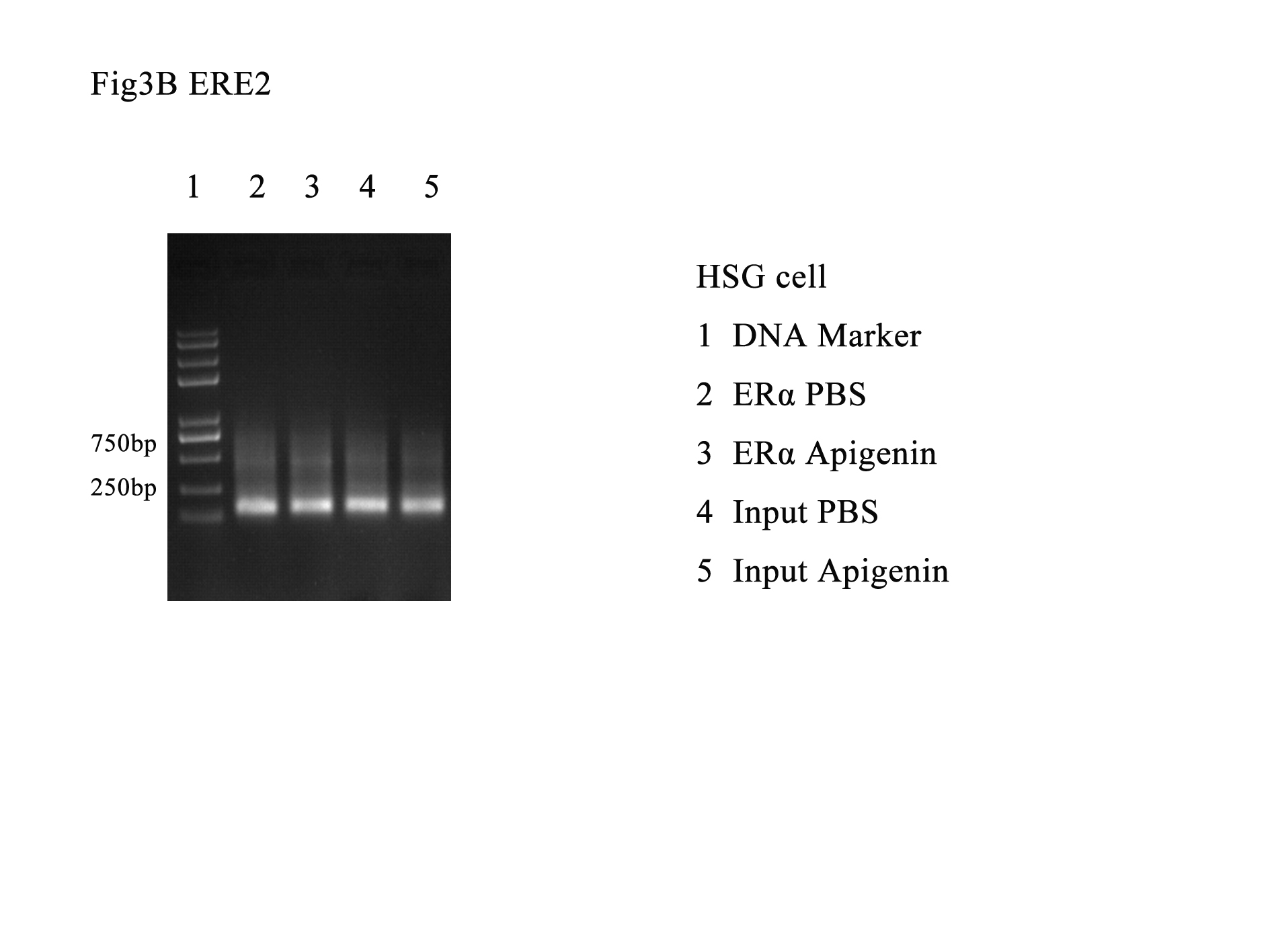

Supplement: Supplementary file 1 [file Image3.JPEG]

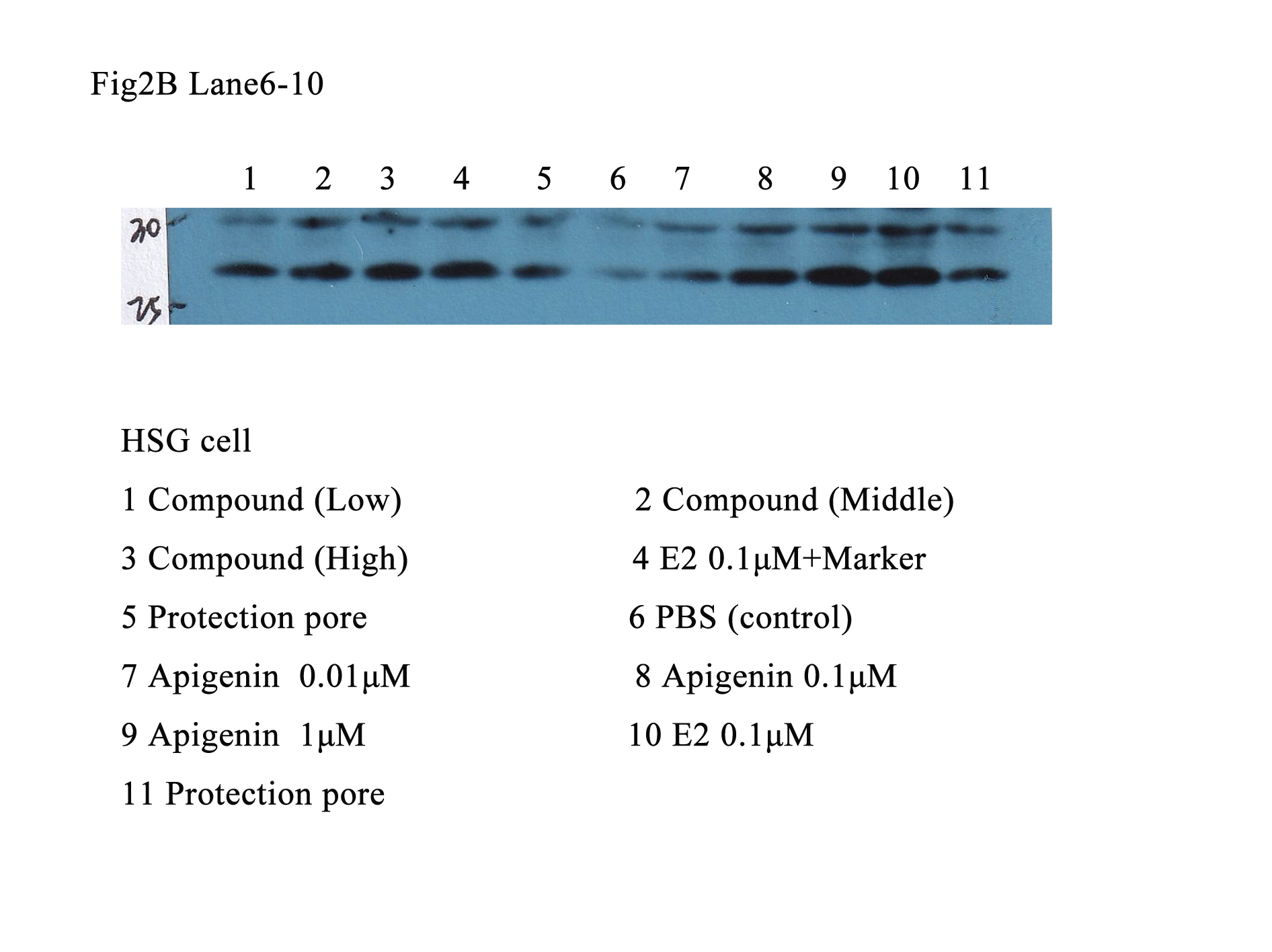

Supplement: Supplementary file 5 [file Image1.JPEG]

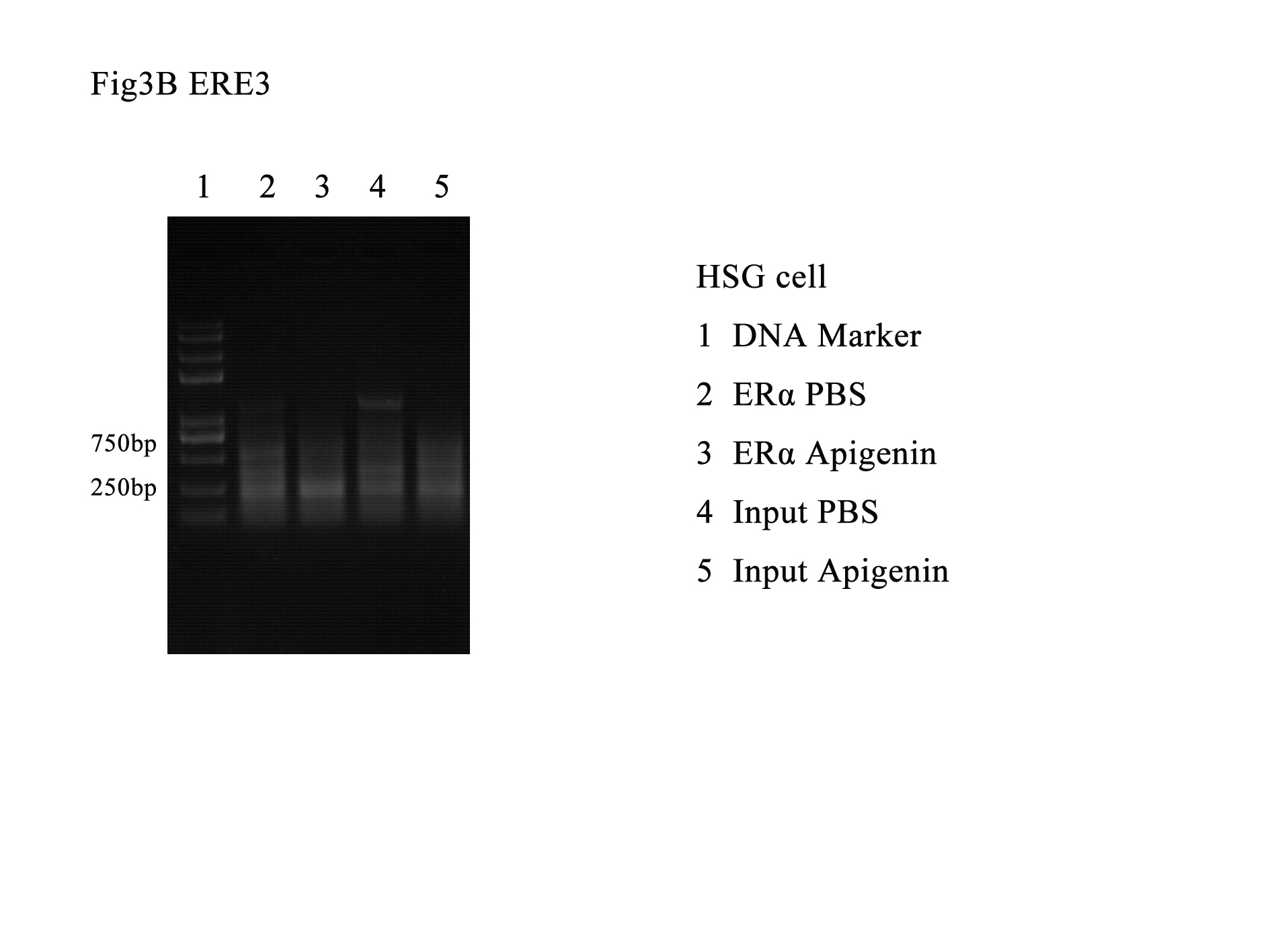

Supplement: Supplementary file 6 [file Image4.JPEG]

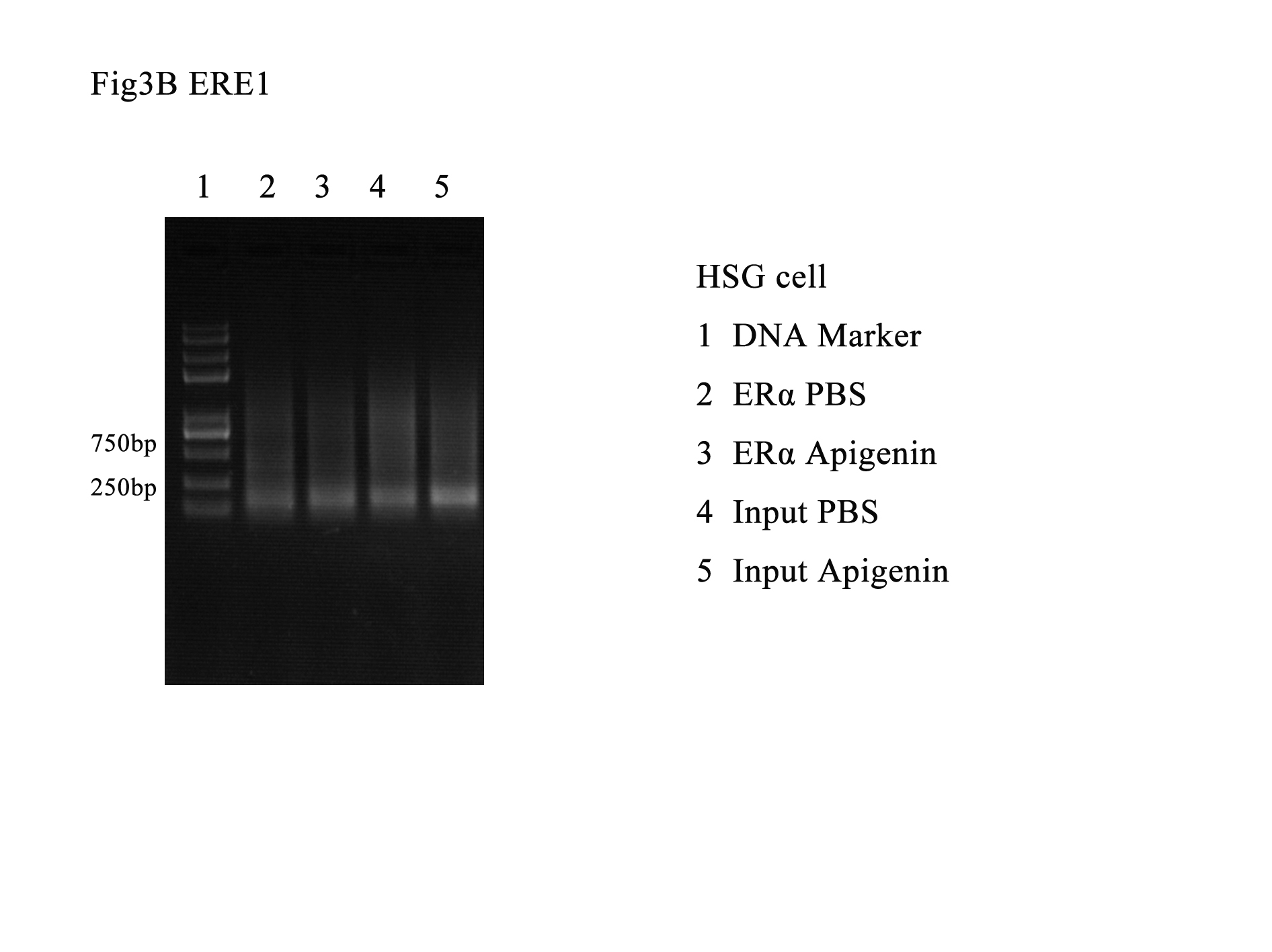

Supplement: Supplementary file 7 [file Image2.JPEG]

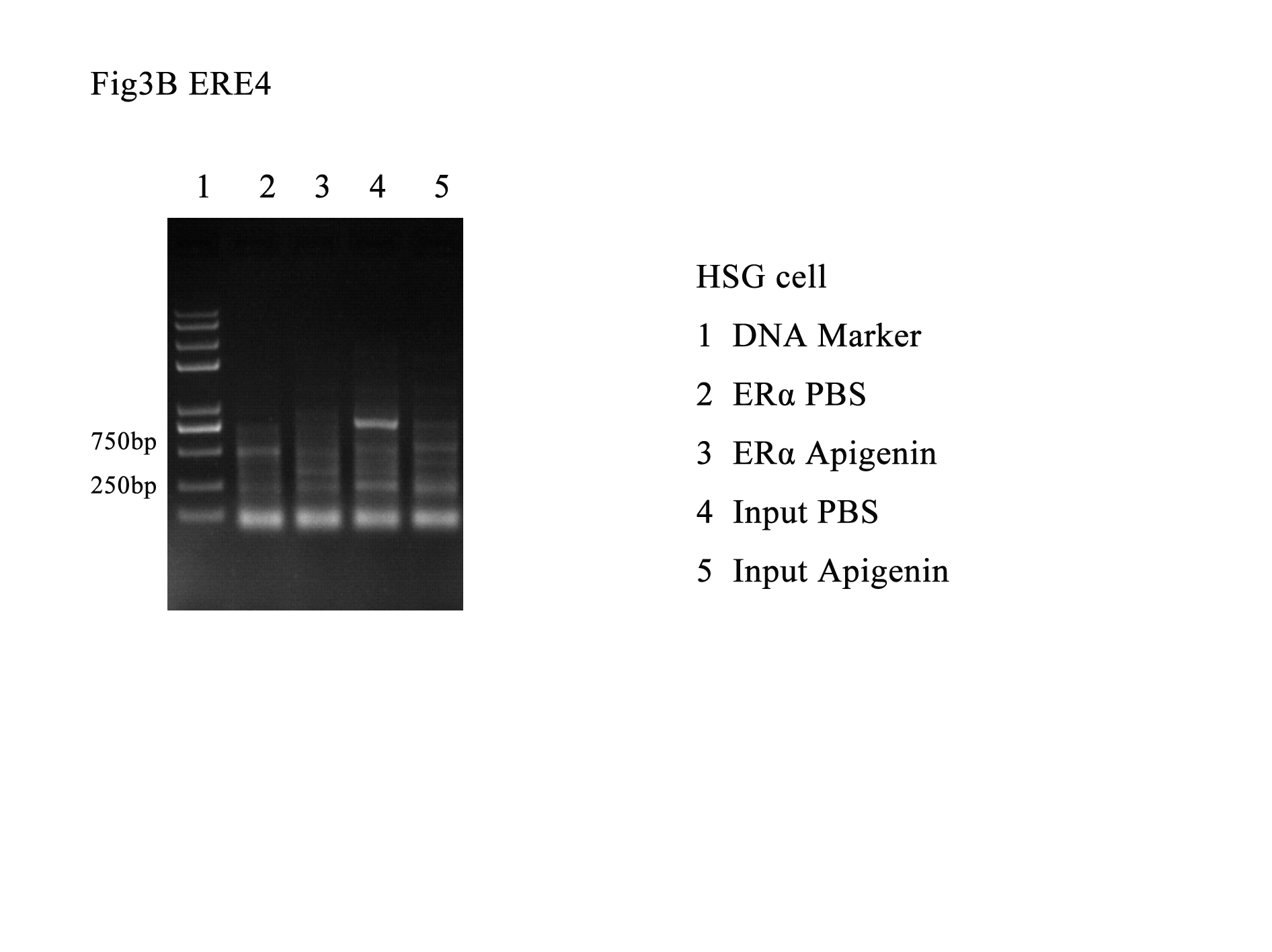

Supplement: Supplementary file 8 [file Image5.JPEG]

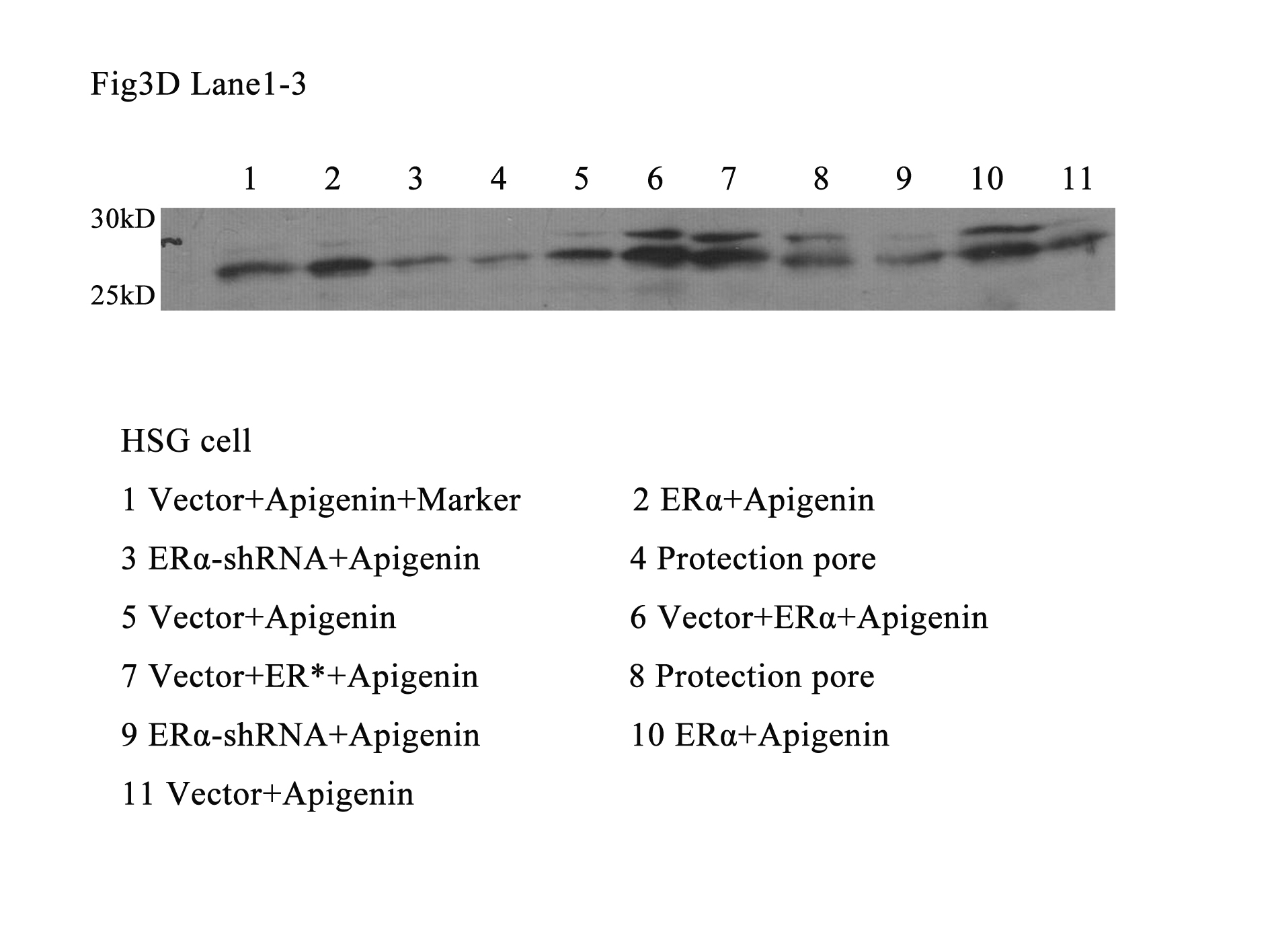

Supplement: Supplementary file 12 [file Image6.JPEG]
